# Adjuvants for peptide-based cancer vaccines

**DOI:** 10.1186/s40425-016-0160-y

**Published:** 2016-09-20

**Authors:** Hiep Khong, Willem W. Overwijk

**Affiliations:** 1 Department of Melanoma Medical Oncology, University of Texas - MD Anderson Cancer Center, South Campus Research Building 1, 1515 Holcombe Blvd, Houston, TX 77030 USA; 2 Immunology program – University of Texas - Graduate School of Biomedical Sciences at Houston, 6767 Bertner Ave, Houston, TX 77030 USA

**Keywords:** Cancer vaccine, Peptide, T cells, Checkpoint, Adjuvant

## Abstract

Cancer therapies based on T cells have shown impressive clinical benefit. In particular, immune checkpoint blockade therapies with anti-CTLA-4 and anti-PD-1/PD-L1 are causing dramatic tumor shrinkage and prolonged patient survival in a variety of cancers. However, many patients do not benefit, possibly due to insufficient spontaneous T cell reactivity against their tumors and/or lacking immune cell infiltration to tumor site. Such tumor-specific T cell responses could be induced through anti-cancer vaccination; but despite great success in animal models, only a few of many cancer vaccine trials have demonstrated robust clinical benefit. One reason for this difference may be the use of potent, effective vaccine adjuvants in animal models, *vs*. the use of safe, but very weak, vaccine adjuvants in clinical trials. As vaccine adjuvants dictate the type and magnitude of the T cell response after vaccination, it is critical to understand how they work to design safe, but also effective, cancer vaccines for clinical use. Here we discuss current insights into the mechanism of action and practical application of vaccine adjuvants, with a focus on peptide-based cancer vaccines.

## Background

The goal of a therapeutic cancer vaccine is to induce the activation and proliferation of T cells, in particular cytotoxic T lymphocytes (CTL), which specifically recognize and kill cancer cells leading to improved therapeutic outcome for the patient. To maximize CTL responses, an ideal vaccine adjuvant must fulfill two major functions. First, it must provide optimal availability of the antigen (Ag, signal 1) by regulating its persistence, location, concentration and presentation by antigen presenting cells (APC). Second, it must enhance the immune response by inducing the expression of co-stimulatory molecules (signal 2) and cytokines (signal 3) by APC [1]. Suboptimal delivery of any of these signals can result in poor T cell numbers and/or function.

## Antigen delivery systems

**Fig. 1:**
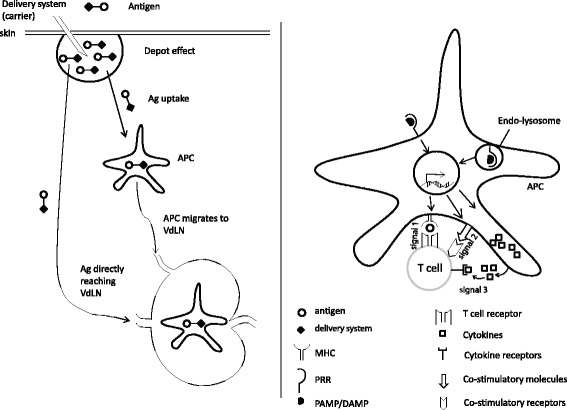
Mechanisms of action of vaccine adjuvant. *Left*, some adjuvants can function as antigen delivery systems to affect the geographical availability of the antigen (signal 1). *Right*, adjuvants also commonly stimulate antigen presenting cells (APC) and induce them to upregulate co-stimulatory molecules such as CD80/CD86 (signal 2) and/or produce cytokines such as IL-12 (signal 3). VdLN: vaccination site-draining lymph node

Antigen delivery systems facilitate signal 1 by different mechanisms. First, they extend Ag presentation time by protecting Ag from degradation by cell-associated serum proteases and peptidases [2]. Second, they enhance the uptake of tiny antigenic peptides by APC by forming them into particles of a size similar to that of pathogens (micrometer or submicrometer size) [3]. Third, some delivery systems can promote the localization of Ag to peripheral draining lymph nodes which increases the chance of encountering draining lymph node-resident APC, resulting in increased Ag-presentation to T cells [4]. Collectively, these mechanisms enhance T cells response number by extending Ag presentation time to be optimal for T cell clonal expansion, effector function and/or memory formation [5, 6] (Fig. 1). Mode of action, the types of responses, and advantage/disadvantages of selected antigen delivery systems are shown in Table 1. Of notice, vaccination can also allow for the delivery of immunodominant or neoantigen epitopes, resulting in enhanced anti-tumor efficacy.

**Table l:** Examples of class I adjuvants (delivery systems)

	Mode of action(s)	Types of response	Pros	Cons
IFA and Montanide formulations	Depot	Ab, Th1, Th2	Widely used for vaccines when antibody production is desired [123].	May not suitable for therapeutic vaccine when cellular response is desired as extended depot will attract CTL to vaccine sites [10].
Aluminum	Depot, inflammasome activation	Ab, Th2	Safety characters are well defined as it is the most widely used adjuvant [124].	Needs to be combined with other adjuvants to induce CTL response in therapeutic vaccines.
Micro/nano particles	Varies, depending on particlenature: increase Ag half-life (via encapsulation, sustained release) delivery Ag to target cells/organs, cellular and Inflammation induction (see text for detail)	Not well defined but size of articles may contribute to types of response: size of 40–50 nm induces stronger T cell response than 20nm or 2000 nm particles ^4^.	Reduce Ag dose, cellular and biological characters are well defined, versatile to be combined with other adjuvants [121].	Rapid clearance in blood and accumulation in filtering organs such as liver and spleen [24]. Need to be combined with immunopotentiators.

Besides signal 1, antigen delivery systems can also deliver signal 2 and 3 by activating the innate immune cells. Aluminum, PLG and polystyrene particles were shown to activate the inflammasome complex in a phagocytosis-dependent manner while carbon nanotubes trigger the complement system (see below). Adjuvants vary in the quality and quantity of signals 1, 2 and 3 they deliver to T cells. These attributes of adjuvants become especially important when using them to vaccinate with antigens that possess very little, if any, inherent adjuvant activity, such as the minimally defined peptide epitopes typically used in peptide vaccines. Here we discuss some adjuvants that are commonly used in peptide-based cancer vaccines.

### Incomplete Freund’s adjuvant (IFA)

IFA is a water-in-oil emulsion, identical to Complete Freund’s Adjuvant (CFA) but without the heat-killed Mycobacteria tuberculosis to avoid acute granulomatous lesions at vaccine sites. It has previously been shown that IFA promotes long-term retention and slow release of emulsified antigen at the inoculation site [7, 8]. Likely as a result of this, IFA induces strong humoral and cellular immune responses. Clinical-grade IFA (Montanide™ oil series, SEPPIC Corp.) has been widely used clinically in experimental peptide and protein-based cancer vaccines [9]. Recently, our group showed that IFA-based peptide vaccines can induce potent cytotoxic CD8 T cell responses in mice, followed by T cell retention, exhaustion and deletion at the vaccination site, due to excessively long-term peptide Ag retention and chronic release by the poorly biodegradable IFA emulsion [10]. Mechanistically, the long-term antigen presentation and consequent T cell recognition and cytokine release at the vaccination site induced chronic tissue inflammation and chemokine production that attracted and retained effector T cells, preventing them from reaching the tumor site. Eventually, persistent antigen stimulation at the vaccination site resulted in T cell exhaustion and Fas/FasL-mediated T cell apoptosis. Of notice, this observation was obtained using vaccines based on minimal epitope-sized short peptides which can be presented by any MHC Class I-positive, nonprofessional APC [11]. In contrast, longer peptides require trimming by DC-specific enzymes to allow efficient binding to MHC Class I molecules, and hence they are presented exclusively by the relatively small population of DC in the context of optimal co-stimulatory molecules and cytokines for efficient T cell priming [12]. Indeed, long peptides emulsified in IFA induced minimal T cell trafficking to vaccine sites and greatly reduced contraction of T cell levels [10]. It is also proposed that long peptides which contain helper T cell epitopes will induce Th response to further enhance the CTL response [12]. However, in 2 separate clinical trials using IFA, separate Th epitopes mixed with short CTL epitopes failed to improve CTL response in patients with metastatic melanoma [13, 14]. This might be due to a difference in the nature of the antigens: virus-derived long peptides containing both Th and CTL epitopes vs. melanocyte self antigen-derived short CTL epitope peptides mixed with short Th epitope peptides. Given the clear benefit of CD4+ T cell responses in the generation and intratumoral function of CD8+ T cells [15, 16], further studies are needed to reconcile this discrepancy. Nevertheless, our preclinical data suggest that prolonged Ag presentation (signal 1), even in the presence of signal 2 and 3, can induce T cell retention, exhaustion and deletion.

### Aluminum adjuvants

Generally referred to as alum, both aluminum hydroxide (Alhydrogel™) and aluminum phosphate (Adjut-phos™) adjuvants are widely used in human vaccines such as those against influenza, tetanus, diphtheria, pertussis, poliomyelitis, and HPV [17]. During vaccine preparation, antigens are adsorbed to preformed aluminum adjuvants, hence their name aluminum-adsorbed vaccines. Aluminum adjuvants are known to promote Th2 responses which make them less suitable for vaccines against intracellular bacteria such as *M. tuberculosis*, which require a Th1-type immune response dominated by IFN-γ [18]. When combined with MPL (a detoxified form of lipopolysaccharide, LPS), a TLR4 agonist, such as in the AS04 adjuvant system (Glaxo SmithKline), alum-based vaccines induce Th1 responses with production of IFN-γ and IgG2a. In 2008, alum adjuvants were found to activate the NALP3 inflammasome in DC [19]. Inflammasome activation leads to the production of proinflammatory cytokines including IL-1β and IL-18 which promote the adaptive cellular (Th1/Th17/Th2) and humoral responses [20]. IL-1β promotes Th1 and Th17 while IL-18 serves as coactivator for other cytokines. In the presence of IL-12 and IL-15, IL-18 contributes to Th1 response via promoting IFN-γ production. In the absence of IL-12, IL-18 induces IL-4 which drives Th2 response [21]. Thus, adjuvants that activate the inflammasome, including alum, can induce different types of T cell response, depending on tissue- or adjuvant-driven cytokine context.

### Micro/nano particles

Micro- and nano-particles are attractive antigen/drug delivery systems because they can combine several desired characteristics. First, the particles protect their cargo from serum/tissue peptidases/proteases and other degrading factors, thus increasing the half-life of encapsulated Ag and immunomodulators in vivo. Second, particles can be engineered to target specific cell types or organs (such as lymph node) [22, 23]. These features help reduce both the drug dose and off-target side effect. For example, it has been shown that Ag encapsulated in poly(lactic-co-glycolic acid) (PLGA) particles induce similar T cell response with a 1000-fold lower dose compared to free Ag [24].

There are two basic ways to engineer particles for enhanced uptake by APC. Passive targeting relies on the size, charge and rigidity of the particle while active targeting is based on added ligands on the particle surface. Vaccine particles with size range from 500 to 2000 nm are preferentially trapped by tissue APC at the injection site (which may then traffic to LN), while 20 to 200 nm particles drain passively to LN where they are taken up by resident APC. Beside their role as Ag/drug carrier, increasing signal 1, micro and nanoparticles can also enhances signals 2 and 3. PLG and polystyrene particles are thought to participate in inflammasome activation by enhancing the IL-1β secretion by DC in a phagocytosis-dependent manner [25]. Carbon nanotube particles, on the other hand, activate the complement system and subsequent inflammatory responses via binding to C1q [26]. Materials used to make micro and nanoparticles include liposomes, synthetic polymers such as polystyrene, poly(lactide-co-glycolide) PLG, poly(lactic acid) PLA, PLGA or natural polymers such as gelatin, collagen and chitosan. The choice of material depends on the desired biocompatibility, half-life, hydrophobicity and polarity. For example, liposome particles are very versatile, allowing combination of Ag and cytokines like IL-2 or GM-CSF, into a single particle to provide better immune response and protection [27]. However, major drawbacks are the rapid clearing from the blood and accumulation in the liver. Coating a liposome with polyethylene glycol (PEG) or other biocompatible polymers can reduce rapid systemic clearing and thus extend its half-life in vivo [28].

To improve the accumulation of a liposome to targeted tissue or organ, its surface can be decorated with receptors (e.g. antibodies) for target cell/tissue ligands and such modified liposomes are called immunoliposomes. Micro- and nanoparticles such as hydrophilic poly(DL-lactide-co-glycolide) microspheres and poly(propylene sulfide) nanoparticles have been designed to target the DC in draining LN [22, 23]. A different approach is to attract DC to the site of vaccine injection. Recent reports showed that incorporating GM-CSF, CpG and tumor antigens in PLG matrices efficiently attracted and stimulated both conventional DC (CD11c + CD11b + and CD11c + CD8a+) and plasmacytoid DC, resulting in superior immune responses (Th1 and CTL) against B16 melanomas in mice [29, 30]. A very high concentration of GM-CSF (3000 ng) prolonged the DC retention in situ, resulting in suboptimal DC trafficking to draining LN and the subsequent inferior T cell priming and protection against tumor. This observation suggests that delivery systems that stimulate the attraction of DC can promote T cell responses, but only if they do not prevent the DC from ultimately reaching the LN where T cell priming typically occurs.

#### The antigen depot: what duration of antigen presentation is optimal?

Our preclinical work with IFA as a vaccine adjuvant suggests that prolonged antigen presentation has multiple detrimental effects on the effector function, tumor localization, and survival of vaccination-induced, tumor-specific T cells [10]. However, extremely short antigen presentation (such as after injection of minimal epitope peptides in saline), especially in the absence of adjuvants to induce signals 2 and 3, can likewise lead to suboptimal or even abortive/tolerogenic T cell activation. We speculate that in successful, natural immune responses, such as those against acute viral infections that are rapidly and completely cleared, the bulk of specific antigen persists for a moderate duration, in the order of a few days [31]. While there is clear evidence that small amounts of antigens can be retained much longer in APC, the initial large wave of antigen that primes the acutewave of T cell effectors that follows within days of acute pathogen exposure is typically gone within a week. By analogy, cancer vaccines with similar kinetics of antigen availability have the best chance of priming a massive wave of tumor-specific CTL. Indeed, we have observed such a bell-shaped curve for T cell response and function after different duration of antigen presentation in vivo (Khong et al., manuscript in preparation). It will be interesting to see whether this is a common principle, and whether this can be harnessed to increase the potency and efficacy of peptide-based cancer vaccines.

## The immunopotentiators

When vaccinologists moved from whole pathogen vaccines (live, attenuated or dead pathogens) to recombinant subunit vaccines for reasons of safety and manufacturing, they learned that these vaccines typically evoked weaker immunity and protection. The discovery of how our body senses pathogens via a family of highly conserved pattern recognition receptors (PRR) called Toll-like receptors (TLR) [32–34] heralded the era of the specific receptor-mediated activation of innate immunity. Since then, other innate immune receptors have been discovered including NOD-like receptors (NLR), C-type lectin receptors and retinoic acid inducible gene (RIG)-I-like receptors (RLR) and most recently cyclic GMP-AMP synthase (cGAS). Within the last decades, numerous adjuvants have been developed to target these innate receptors. Signaling mechanisms of these receptors have been thoroughly discussed elsewhere [35–38]; here we focus on the adjuvants that target these receptors, in particular those that have entered clinical trials of cancer vaccines. Some notable examples of immunopotentiators and their stages of development are listed in Table 2.

**Table 2:** Examples of class 2 adjuvants (immunopotentiators)

	Receptor	Target cells	Stage of development (not comprehensive) in cancer vaccine
“stepping on the gas”			
Pam_3_CSK_4_	TLR2	DC, M$, lymphocytes	Preclinical
Poly-ICLC	TLR3	cDC, M$, epithelial cells	Several clinical trials for different cancers.
MPLA	TLR4	cDC, M$, epithelial cells, fibroblasts	Clinical trial phase 2
Imiquimod	TLR7/8	pDC, B cells, M$, monocytes	Clinically approved for treating basal cell carcinoma. Multiple clinical trials in combination with vaccine for different cancers.
CpG	TLR9	pDc, B cells	Multiple clinical trials
IL-2	IL-2Ra/p/y	T, B and NK cells	Clinically approved for treating renal carcinoma and melanoma. Multiple clinical trials in combination with vaccine for different cancers.
GM-CSF	GM-CSFR	many	Multiple clinical trials in combination with vaccine and checkpoint blockades for different cancers.
IFNs	IFNR	many	Multiple clinical trials
CDNs	STING	many	Preclinical
“releasing the brake”			
a-PD1 Ab	PD-1	T, B and NK cells	Clinically approved for different cancers.
a-CTLA4 Ab	CTLA-4	T cells	Clinically approved for melanoma, under multiple clinical trials for different cancers.

### Adjuvants targeting toll-like receptors

#### TLR2 agonists

TLR2 is expressed on the surface of different immune cells like DC, macrophages and lymphocytes and recognizes bacterial lipopeptides. Upon engaging its ligands, TLR2 activates NF-kB via the MYD88 signaling pathway. There are two common strategies to engage TLR-2 through vaccines: conjugating the antigen to bacterial lipopeptides or to palmitic acid. Bacterial lipopeptide MALP-2 and its synthetic analogues like Pam_2_Cys and Pam_3_Cys are most frequently used. The peptide-lipopeptide construct were shown to induce DC maturation, pro-inflammatory cytokine (IL-12, TNF-α, IFN-γ) secretion, B cell activation and enhanced CTL responses [39]. Most current clinical trials of TLR-2 based adjuvants are for vaccination against infectious diseases such as HIV, HBV and Lyme disease. In 2014, vaccine using TLR-2 ligand (Pam_3_CSK_4_) conjugated with long synthetic peptide showed very promising results in a preclinical melanoma model [40]. Interestingly, Pam_3_CSK_4_-peptide conjugate, but not the mixture of Pam_3_CSK_4_ with peptide, induced robust T cell response and protection against tumor. This is in line with the cis-activation model showed by Desch et al. [41], which essentially posits that signal 1 and 2 should be delivered by same APC for optimal T cell priming.

#### TLR3 agonists

TLR3 is expressed in the endosomal compartment of conventional dendritic cells (cDC), macrophages and on the surface membrane of non-immune cells like epithelial cells [42]. TLR3 is activated by double-stranded RNA or its synthetic analog polyinosine-polycytidylic acid (poly I:C) [43]. TLR3 does not use the MyD88 signaling pathway but triggers TRIF signaling leading to activation of NF-kB, MAP kinases and IRF3, which in turn induce the production of inflammatory cytokines, type 1 interferons (IFNs) and the subsequent upregulation of costimulatory molecules [44].

Poly I:C can enhance antigen cross-presentation by DC to CD8 T cells. Because of its rapid degradation by serum nucleases in primates, poly I:C has limited anti-tumor efficacy in humans [39]. Therefore, more stable derivatives of poly I:C were made, including poly ICLC (known as Hiltonol) and poly I:C_12_U [45]. In a phase 1 ovarian cancer trial, addition of poly ICLC to a vaccine consisting of NY-ESO1 long overlapping peptides in IFA dramatically induced rapid and efficient CD4 and CD8 T cell responses, compared to the vaccine alone [46]. A recent study in monkeys showed that poly ICLC in combination with agonistic CD40 antibody significantly enhanced both CD4 and CD8 responses compared to either adjuvant alone [47]. This is some of the first primate data confirming the multitude of mouse studies that indicated strong synergy when different classes of immunopotentiators are used together in vaccine adjuvants [10, 48, 49]. I:C_12_U and poly ICLC have entered clinical trials for other cancer including glioma, melanoma, carcinoma (poly ICLC) and HER-2 positive breast cancer [39].

#### TLR4 agonists

TLR4 is expressed on the surface of immune cells including cDC and macrophages as well as non-immune cells such as fibroblasts and epithelial cells. Triggering TLR4 will activate both MyD88 and TRIF dependent pathways leading to NF-kB and IRF3/7 activation. TLR4 activation strongly promotes Th1 response through IL-12p70 induction [50]. Due to its high toxicity, LPS has been replaced by the less toxic derivative, monophosphoryl lipid A (MPLA), as vaccine adjuvant. The adjuvanticity of MPLA has been studied extensively in several clinical trials [39]. MPLA is used in combination with aluminum (AS04) to skew the typical Th2 response induced by alum to a Th1 response [51]. MPL as a vaccine adjuvant, in combination with tumor antigens, has entered into several clinical trials for melanoma, lung, and prostate cancer [52–54].

#### TLR7/8 agonists

Localizing within the endosomal compartments, both TLR7 and 8 can recognize single stranded (ss) RNA as they are structurally related [42]. In human, TLR7 is predominately expressed in plasmacytoid dendritic cells (pDC) and to a lesser extent in B cells and monocytes/macrophages while TLR8 is mainly expressed in monocytes/macrophages and cDC [55]. TLR7/8 signal through the MyD88 pathway leading to upregulation of co-stimulatory molecules (CD80/86, CD40), production of cytokines (IFN-α, TNF-α, IL-12) and migration of DC from skin to lymph nodes. TLR8 is expressed, while TLR7 is not, on the important BDCA3+ cDC subset that is most potently responsible for cross-priming of CD8+ T cells [56], and thus preferential TLR7 agonists may exert weaker adjuvant activity than TLR8 or TLR7/8 agonists when used in CD8+ T cell-inducing vaccines. TLR7/8 can also activate B cells to produce antibody and cytokines such as IL-6 and TNF-α, and T cells to proliferate and produce cytokines including IFN-γ and IL-2 [57, 58]. TLR7/8 can be activated by synthetic imidazoquinolines including imiquimod (mostly acts on TLR7) and resiquimod (TLR7 and 8). Imiquimod (Aldara cream) has been approved to treat basal cell carcinoma and genital warts [59, 60]. Several clinical trials of imiquimod as vaccine adjuvant in different cancers including chronic myeloid leukemia (CML), vulval intraepithelial neoplasia (VIN), prostate cancer and melanoma have been conducted [61–64]. Overall, all vaccines induced both humoral and cellular responses in a major fraction of patients. In vaccinated patients with VIN, infiltration of both CD4 and CD8 T cells into tumor sites was shown to correlate with tumor clearance [62].

#### TLR9 agonists

TLR9 is expressed by human B cells and pDC and localizes in endo-lysosomal compartment [42]. Its role is to detect unmethylated CpG motifs which are often found in bacterial, but not host cell DNA. Upon activation, TLR9 induces production of pro-inflammatory and Th1 cytokines (such as IL-12) by APC. There are 3 classes of synthetic CpG oligonucleotides (ODN) being used in preclinical and clinical studies. CpG A is a mix of phosphodiester/phosphorothioate backbone with palindromic sequences and poly G tail, and is a potent pDC activator and IFNα inducer [65]. CpG B only has phosphorothioate backbone. CpG B strongly activates B cells and promotes pDC and monocyte maturation [66]. CpG C is a hybrid of the two above [67]. CpG has been used in clinical trials of therapeutic cancer vaccines against melanoma, breast/lung/ovarian cancers, sarcoma and glioblastoma [68–72]. Overall, the vaccines induced both humoral and cellular responses, but clinical benefit remained uncommon.

### STING agonist

In 2006, TLR-independent antiviral responses (i.e. type 1 interferon induction) were shown to be induced by double stranded (ds) DNA in the cytosol [73]. Later, dsDNA was found to activate the transcription factor NF-kB and IRF3 via an endoplasmic reticulum adaptor called STING (stimulator of interferon genes) [74]. In 2013, the receptor for cytosolic DNA, the cylic GMP-AMP synthase or cGAS, was discovered [75]. Upon binding to cytosolic DNA, cGAS catalyzes the synthesis of cGAMP which in turns binds to and activates the adaptor protein STING. Recent results indicate that spontaneous T cell priming against tumor antigen requires STING-dependent type I IFN induction [76]. Very promising results from preclinical studies with STING agonists injected directly into tumors in the aggressive B16 melanoma model had led to high excitement for their application in clinical trials [77]. Recent results also indicate that STING agonists can function as adjuvant in a setting of whole-cell tumor cell vaccine [78]. It will be interesting to see how STING agonists compare to TLR agonists as adjuvants for peptide vaccines in animal models and clinical trials, and whether their combined use offers additional benefit, given their different intracellular signaling pathways.

### Cytokines as adjuvants

#### IL-2

The most notable cytokine which has been extensively used for immunotherapy is IL-2. IL-2 was initially described as a T cell growth factor (TCGF) responsible for the clonal expansion, differentiation and survival of T cells [79], and later of activated B cells and natural killer (NK) cells as well [80, 81]. Although CD4 T cells are the major source of IL-2 in vivo, CD8 T cells, NK cells and DC can also produce IL-2 [82–85]. IL-2 was FDA-approved for the therapy of metastatic renal cell carcinoma in 1992 and metastatic melanoma in 1998 [86, 87]. IL-2 mediates anti-tumor activity by activating tumor-specific T cells and NK cells. In mice, addition of IL-2 to experimental cancer vaccines can greatly increase the therapeutic efficacy [10, 48]. IL-15 signals through the same IL-2 Rβγ complex also used by IL-2, and can also promote peptide-induced T cell proliferation, especially in T cells with low-affinity TCRs [88]. In patients with melanoma, addition of an experimental gp100 peptide/IFA vaccine to IL-2 gave a higher clinical response rate than observed in patients receiving IL-2 alone, and also higher than previously observed for gp100 peptide vaccine alone, suggesting IL-2 can also function as a vaccine adjuvant in humans [89]. However, IL-2 can also expand immunosuppressive regulatory T cells (Treg) which may dampen the immune response or anti-tumor efficacy [90]. Because Treg express both IL-2Rα and IL-2Rβγ while CTL express only the latter, blocking IL-2Rα when using IL-2 preferentially expands CTL [91]. Recently, a mutant form of IL-2 (IL-2 mutein) was reported to have higher antitumor efficacy with reduced proliferation induction on Treg, possibly thanks to preferential binding to IL-2Rβγ but not IL-2Rα [92]. Similarly, IL-2 pre-complexed with IL-2-specific antibodies, and IL-2 covalently modified with polyethylene glycol have shown selective binding to IL-2Rβγ but not IL-2Rα, favoring selective effects on CD8+ T cells [93, 94]. If these modifications also lower the toxicity of IL-2, which may be partly mediated by IL-2Rα, these IL-2-based compounds may make a comeback in cancer immunotherapy, including as vaccine adjuvants [91].

#### Granulocyte-macrophage colony stimulating factor (GM-CSF)

GM-CSF is a cytokine used as a cancer vaccine adjuvant, sometimes with success. GM-CSF can be produced by many cell types including myeloid cells, lymphocytes, fibroblast, endothelial/epithelial/mesothelial cells and certain tumor cells [95]. The production of GM-CSF is induced by bacterial toxin and inflammatory cytokines such as IL-1, IL-6, and TNF-α [96]. GM-CSF receptor is found on myeloid cells and non-hematopoietic cells such as endothelial cells. In vaccine settings, GM-CSF has been shown to initiate the recruitment and maturation of DC as well as activation of macrophages, neutrophils, and NK cells, indicating that it is a potential vaccine adjuvant [97, 98]. Combination of GVAX (irradiated tumor cell expressing GM-CSF) with anti-CTLA-4 and anti-PD-1 checkpoint blockade was very promising in preclinical studies, leading to the first clinical trials of checkpoint blockade in patients with cancer. Recombinant GM-CSF has been used in peptide vaccine trials in mouse and man, where it has had varying success in raising T cell responses. This may be partially due to a balance between pro- and anti-inflammatory properties of GM-CSF depending on its dose [29]. In addition, there appear to be complex interactions between GM-CSF and other factors in the tumor-conditioned microenvironment that influence its ability to either enhance or reduce vaccine-induced T cell responses [99–102]. Several positive peptide/protein vaccine trials have incorporated GM-CSF [13, 103]; however due to the lack of a vaccine arm without GM-CSF, its exact impact on clinical outcome remains unknown [104, 105].

#### Interferons (IFNs)

IFNs are of great interest for adjuvant development, owing to their pleiotropic effect on different immune cells such as DC, B cells and T cells as well as non-immune cells. IFN-α and IFN-β promote DC maturation, including the up-regulation of MHC and costimulatory molecules. In virus-infected cells, type I IFNs prevent virus replication by halting transcriptional and translational machineries, accelerating RNA degradation by inducing RNase L and inducing apoptosis [106]. IFN-α and pegylated IFN-α have been approved for advanced renal cell carcinoma and chronic hepatitis C treatment, respectively, and both are given after surgical resection of primary melanoma to reduce the chance of recurrence [107]. Preclinical studies showed direct adjuvant efficacy of type I IFN in a peptide-based anti-melanoma vaccine, where it promoted T cell numbers, longevity and effector function, resulting in improved tumor control [108]. In contrast to type I IFN, IFN-γ (the sole type II IFN) is typically only produced by specialized immune cells including T cells, NK cells and NKT cells [109]. Recombinant IFN-γ (or genetically engineered IFN-γ1b) is approved to treat chronic granulomatous disease [110]. In cancer immunotherapy, a phase III clinical trial combining chemotherapy with IFN-γ for patients with advanced ovarian and peritoneal carcinomas was terminated due to serious adverse effects [111].

#### Lessons learned from a few successful peptide-based cancer vaccine clinical trials

A vaccine comprised of long peptide from HPV-16 viral oncoproteins E6 and E7 emulsified in IFA was shown to be very effective in treating vulvar intraepithelial neoplasia, a precancerous condition in HPV-16 positive women [112]. The overall clinical response was 79 % while complete response was 47 %, after 2 years of follow-up. This remarkable result with an IFA-based peptide vaccine was consistent with our findings that long peptides did not cause severe sequestration of T cells at the vaccination site as discussed above. In a phase 3 trial for patients with advanced melanoma, combination of IL-2 with short gp100 (209–217) peptide emulsified in IFA resulted in a modest but significant improvement of overall clinical responses, progression-free survival and overall survival, compared to IL-2 treatment alone [89]. Based on some of the preclinical results with IFA discussed above, a less persistent, and therefore less T cell sequestering, vaccine formulation might result in more dramatic synergy with IL-2. Indeed, given new insights into the nature of tumor antigens (short vs. long peptides, as well as mutated vs. non-mutated antigens) and adjuvants, there is ample opportunity to design new, more effective cancer vaccines. A clinical trials in renal cell cancer with the multiple peptide-based, GM-CSF-adjuvanted, water-formulated IMA091 vaccine showed that the breadth of CTL response significantly associated with clinical benefit, perhaps by limiting antigen-loss escape mechanisms [104, 113]. Taken together, results from these clinical studies underscore the importance of the nature and delivery of target antigens, and the provision of the right adjuvant.

#### Cancer vaccines and T cell checkpoint blockade

While blockade of CTLA-4 and PD-1 T cell checkpoints shows strong activity in a variety of cancers, many patients do not respond, likely due to insufficient spontaneous anti-tumor T cell immunity (a lack of tumor reactive T cells and/or poor T cell infiltration into the tumor). Vaccination can enhance tumor-specific immunity, and vaccination is therefore a prime candidate for combination with checkpoint blockade therapy. Interestingly, the 676-patient study that led to FDA approval of anti-CTLA-4 revealed that concurrent vaccination with gp100 peptide vaccine in IFA did not enhance therapeutic efficacy, and in fact modestly but significantly decreased overall response rate and disease control rate through an unknown mechanism [114]. This has led to uncertainty about whether and how to combine vaccination with checkpoint blockade, hampering efforts to improve overall response rates in melanoma and especially in other, less immunogenic cancers. When modeled in mice, we indeed observe that gp100/IFA vaccination does not synergize with CTLA-4 or PD-1, and that this effect is due to T cell entrapment, even of anti-CTLA-4 therapy-induced T cells, at the gp100/IFA vaccination site. Nevertheless, by choosing different vaccine formulations, great synergy between peptide vaccine and checkpoint blockade can be achieved (unpublished results). Other preclinical work also indicates synergy between checkpoint blockade and other classes of non-persistent vaccines, opening the possibility that vaccines that do not induce excessive T cell sequestration may combine well with checkpoint blockade therapy [78, 99].

#### The need for combining different adjuvants into a single vaccine

Much preclinical work suggests that combining different adjuvants is needed to induce a strong anti-tumor immune response [115]. Accumulated evidence has shown that CD40 signaling synergizes with almost all TLR ligand inducing far better cellular and humoral responses than that of each individual adjuvant [116, 117]. Several groups have shown that almost all TLR agonists synergize with CD40 signaling to enhance CTL expansion and function, in part by inducing the co-stimulatory molecule CD70 on DC [118]. We found that adding IL-2 to a TLR7 agonist/CD40 agonist combination further enhanced CD8 T cell peak effector and memory response, and anti-tumor efficacy [10]. Second, some adjuvants may possess both desired and undesired adjuvant properties. By combining with other adjuvants, the immune response can be skewed toward favorable one, as in the above-mentioned example of alum combined with MPL which is used in HPV vaccine and HBV vaccine to promote Th1 response [119]. A major obstacle to successful translation of these long-known preclinical findings is the observable paucity of clinical trials where multiple pharmaceutical companies combine their respective promising, potent agents to create a truly powerful cancer vaccine. This limitation is slowly beginning to be addressed by the initiation of co-development agreements between companies, as well as by the development of multiple synergistic adjuvants within single companies. Thus, clinical trials of cancer vaccines consisting of multiple antigens formulated in adjuvants consisting of short-lived depots with multiple classes of synergistic immunostimulatory molecules may finally become a reality.

#### Adjuvant-free peptide vaccine

There is an emerging new trend of adjuvant-free vaccine that uses self- assembling peptides. Such peptides were constructed to have a domain which helps them assemble into nanofiber structure [120]. Preclinical studies using mouse model showed self-assembling peptides could elicit humoral as well as cellular responses [120–122]. The humoral response was shown to be T cell independent, possibly due to cross-liking of repetitive epitopes of nanofiber peptides to B cell receptors. Yet, mechanisms of how self-assembling peptides can trigger cellular responses remain undefined. Nevertheless, we anticipate that while self-assembling peptide cancer vaccines can possibly bypass the need for a separate antigen delivery system, they will still needs immunopotentiators to optimally activate T cells as well as protecting them from tumor suppressive mechanisms to ultimately maximize therapeutic vaccine efficacy.

## Conclusion

Cancer vaccines are attracting new interest as combination partners with other immunotherapies, in particular T cell checkpoint blockade approaches. A detailed understanding of the mechanism of action of anti-cancer vaccination is critical for the design of potent vaccine approaches that induce robust T cell responses. Vaccine adjuvants are a major, required component of successful vaccines, and several novel adjuvants are now making their appearance in the clinic, bridging the wide gap between preclinical and clinical cancer vaccine formulations. This translational effort is further guided by early signs of success in a few clinical trials. The hope is that these new cancer vaccines, alone or in combination with CTLA-4 and PD-1 checkpoint blockade, will increase the duration and quality of life of patients with cancer.

## Abbreviations

APC: Antigen presenting cells
cDC: Conventional dendritic cells
CDN: Cyclic dinucleotide
CFA: Complete Freund’s adjuvant
cGAS: Cyclic GMP-AMP synthase
CTL: Cytotoxic T lymphocytes
CTLA-4: Cytotoxic T-lymphocyte-associated protein 4
DAMP: Damage associated molecular patterns
GM-CSF: Granulocyte macrophage colony- stimulating factor
HBV: Hepatitis C virus
HPV: Human papilloma virus
IFA: Incomplete Freund’s adjuvant
IFN: Interferons
IL: Interleukin
IRF: Interferon response factors
MPL: Monophospholipid A
NK: Natural killer cells
Mɸ: Macrophage
PAMP: Pathogen associated molecular patterns
PD-1: Programmed cell death protein 1
pDC: Plasmacytoid dendritic cells
PLA: Poly(lactic acid)
PLG: Poly(lactide-co-glycolide)
PLGA: Poly(lactic-co-glycolic acid)
poly IC: Polyinosine-polycytidylic acid
PPR: Pattern recognition receptors
RLR: C-type lectin receptors and retinoic acid inducible gene (RIG)-I-like receptors
STING: Stimulator of interferon genes
Th (1,2,17): Helper T cells (type 1, 2, 17)
TLR: Toll-like receptors
TNF: Tumor necrosis factors
Treg: Regulatory T cells
TRIF: TIR-domain-containing adapter-inducing interferon-β
VdLN: Vaccination site-draining lymph node
